# Glial and Neuronal Glutamate Transporters Differ in the Na^+^ Requirements for Activation of the Substrate-Independent Anion Conductance

**DOI:** 10.3389/fnmol.2017.00150

**Published:** 2017-05-29

**Authors:** Christopher B. Divito, Jenna E. Borowski, Nathan G. Glasgow, Aneysis D. Gonzalez-Suarez, Delany Torres-Salazar, Jon W. Johnson, Susan G. Amara

**Affiliations:** ^1^Center for Neuroscience, Department of Neurobiology, University of PittsburghPittsburgh, PA, United States; ^2^Center for Neuroscience, Department of Neuroscience, University of PittsburghPittsburgh, PA, United States; ^3^Laboratory of Cellular and Molecular Neurobiology, National Institute of Mental Health, National Institutes of HealthBethesda, MD, United States

**Keywords:** neurotransmitter transporters, chloride channels, electrophysiology, glutamate, excitatory amino acid transporter

## Abstract

Excitatory amino acid transporters (EAATs) are secondary active transporters of L-glutamate and L- or D-aspartate. These carriers also mediate a thermodynamically uncoupled anion conductance that is gated by Na^+^ and substrate binding. The activation of the anion channel by binding of Na^+^ alone, however, has only been demonstrated for mammalian EAAC1 (EAAT3) and EAAT4. To date, no difference has been observed for the substrate dependence of anion channel gating between the glial, EAAT1 and EAAT2, and the neuronal isoforms EAAT3, EAAT4 and EAAT5. Here we describe a difference in the Na^+^-dependence of anion channel gating between glial and neuronal isoforms. Chloride flux through transporters without glutamate binding has previously been described as substrate-independent or “leak” channel activity. Choline or N-methyl-D-glucamine replacement of external Na^+^ ions significantly reduced or abolished substrate-independent EAAT channel activity in EAAT3 and EAAT4 yet has no effect on EAAT1 or EAAT2. The interaction of Na^+^ with the neuronal carrier isoforms was concentration dependent, consistent with previous data. The presence of substrate and Na^+^-independent open states in the glial EAAT isoforms is a novel finding in the field of EAAT function. Our results reveal an important divergence in anion channel function between glial and neuronal glutamate transporters and highlight new potential roles for the EAAT-associated anion channel activity based on transporter expression and localization in the central nervous system.

## Introduction

Excitatory amino acid transporters (EAATs) are a family of five carriers expressed in the central nervous system (Danbolt, 2001). EAAT1 and 2 are primarily located in glia, EAAT3 is ubiquitously expressed in neurons, EAAT4 is predominantly expressed in cerebellar Purkinje cells (Kanai and Hediger, 1992; Arriza et al., 1994; Fairman et al., 1995; Dehnes et al., 1998; Huang et al., 2004), and EAAT5 is exclusively expressed in neurons of the retina (Arriza et al., 1997). All members of the EAAT family function as secondary active transporters mediating the translocation of 1 L-glutamate molecule coupled to the co-transport of 3 Na^+^, 1 H^+^, and the counter-transport of 1 K^+^ ion (Zerangue and Kavanaugh, 1996; Watzke et al., 2000). This coupling allows the net inward movement of two positive charges with each glutamate translocated into the cytoplasm.

EAATs also possess a thermodynamically uncoupled anion conductance (Fairman et al., 1995; Wadiche et al., 1995b), which displays a permeability sequence generally following SCN^−^ > ClO_4_^−^ > NO_3_^−^ > I^−^ > Br^−^ > Cl^−^ (Wadiche et al., 1995a; Billups et al., 1996; Wadiche and Kavanaugh, 1998). The anion channel is initially gated by the binding of Na^+^ with a subsequent increase in channel open probability upon glutamate binding (Schwartz and Tachibana, 1990; Watzke et al., 2001; Melzer et al., 2003; Torres-Salazar and Fahlke, 2006, 2007; Kovermann et al., 2010; Machtens et al., 2011a; Schneider et al., 2014). The EAATs are trimeric proteins of three identical subunits (Yernool et al., 2004) and each monomer is capable of binding and transporting glutamate, as well as permeating anions (Grewer et al., 2005; Koch et al., 2007; Leary et al., 2007). Several groups have proposed that a tight structural coupling controls the equilibrium between the substrate transport cycle and the opening of the anion channel (Borre et al., 2002; Machtens et al., 2015; Torres-Salazar et al., 2015). Moreover, it has been recently shown that the opening of the channel is facilitated by a lateral movement of the core domain from intermediate states of the protein, which favor the formation of an aqueous and anion selective pathway between the transport and the trimerization domain (Verdon and Boudker, 2012; Cater et al., 2014; Machtens et al., 2015). However, the precise mechanism of anion channel gating is still not well understood. A physiological role for the EAAT-mediated anion conductance has yet to be elucidated for all isoforms. Anion flux through EAAT5 has been shown to regulate cellular activity in the retina (Picaud et al., 1995) and influence the membrane potential and synaptic release rates of retinal bipolar cells (Veruki et al., 2006). Additional data have substantiated that EAAT5 is a ligand gated ionotropic receptor (Arriza et al., 1997; Wersinger et al., 2006; Gameiro et al., 2011) and support the need to investigate the roles of these anion channels in other EAAT isoforms.

To date, only minor differences have been found between the five mammalian EAAT isoforms such as the binding affinity for glutamate (2.5 μM for EAAT4 to 97 μM for EAAT2), the kinetics of transport and the ratio of substrate transport vs. anion permeation (Arriza et al., 1994; Seal and Amara, 1999; Mim et al., 2005; Torres-Salazar and Fahlke, 2007). Minor differences in the channel function of the various EAAT isoforms (Otis and Jahr, 1998; Bergles et al., 2002) have not been attributed to any residue differences or structural disparities between EAAT isoforms and are not known to mediate any significant difference in the regulation of glutamate in the synapse and beyond. Moreover, no differences in intrinsic EAAT function discovered thus far can be attributed to the difference in cell-type expression *in vivo*. Here we report that glial EAAT1 and EAAT2 mediate substrate and Na^+^-independent conducting states unlike the classically Na^+^-dependent neuronal isoforms EAAT3 and EAAT4. This is the first report to segregate EAAT anion channel gating mechanism by isoform-specific, cell-type expression and ushers in a new avenue to study the relationship between structure and function of the various EAAT isoforms.

## Materials and Methods

### Transfections in Mammalian Cell Lines and cRNA Injections in *Xenopus* Oocytes

Human EAAT1 (hEAAT1), hEAAT2, hEAAT3, or rat EAAT4 (rEAAT4) were subcloned into pcDNA3.1 (Invitrogen) using Kpn1 and XbaI restriction sites. rEAAT4 was used rather than hEAAT4 due to significantly better expression in oocytes and only minor differences in primary sequence. tsA201 cells (European Collection of Authenticated Cell Cultures, ECACC) were transfected using Lipofectamine 2000 (Invitrogen) or Fugene 6 (Promega) and incubated in DMEM with 10% FBS and pen/strep. pEGFP was used as a co-tranfection marker for electrophysiological recordings. One day before recording, cells were trypsinized and plated onto 12 mm coverslips for whole-cell patch clamp experiments.

For expression in *Xenopus* oocytes hEAAT1, hEAAT2, hEAAT3 and rEAAT4 were subcloned into pOTV using Kpn1 and XbaI restriction sites as previously described (Arriza et al., 1994). Constructs were linearized with either SmaI (EAAT1–3) or BamHI (EAAT4). cRNA was made using mMessage mMachine T7 kit (Ambion). Fifty microliter injection (10 ng total RNA) was delivered to each oocyte using a Nanoliter 2000 injection system (WPI). After injection, oocytes were incubated at 18°C for 2 to 4 days in 96 mM NaCl, 2 mM KCl, 0.3 mM CaCl_2_, 1.8 mM MgCl_2_, and 5 mM HEPES, pH 7.4 (ND96) containing 50 μg/ml pen/strep, 50 μg/ml of gentamycin, and 50 μM Na^+^ pyruvate.

### Radiolabeled Glutamate Transport Assays

Radiolabeled uptake assays were performed 2 to 3 days after injection of cRNA. Oocytes were pre-incubated for 4 h in either 96 mM NaNO_3_, 2 mM KNO_3_, 0.3 mM CaCl_2_, 1.8 mM MgCl_2_, and 5 mM HEPES, pH 7.4 (hereafter NaNO_3_ solution); 96 mM NMDG-NO_3_, 2 mM KNO_3_, 0.3 mM CaCl_2_, 1.8 mM MgCl_2_, and 5 mM HEPES, pH 7.4 (N-methyl-D-glucamine (NMDG^+^) solution); or 96 mM choline hydroxide, 96 mM HNO_3_, 2 mM KNO_3_, 0.3 mM CaCl_2_, 1.8 mM MgCl_2_ and 5 mM HEPES, pH 7.4 (hereafter ChNO_3_ solution) prior to transport assays. Oocytes were washed with either NaNO_3_ solution, NMDG-NO_3_ solution or ChNO_3_ solution and incubated with 10 μM glutamate +200 nM 3, 4-^3^H-L-glutamate (Perkin Elmer) for 10 min in the appropriate buffers. All oocytes were washed three times with ice cold NaNO_3_ solution to stop transport activity after glutamate incubations and were lysed with 0.1 N NaOH and 1% SDS before liquid scintillation counting was used to record accumulation of tritiated glutamate.

### Two-Electrode Voltage Clamp Recordings

Two electrode voltage clamp recordings were performed using a Geneclamp 500 (Molecular Devices) on stage V-VI oocytes 2 days after injection of cRNA constructs. Holding potential was set to −60 mV and voltage command jumps were performed to voltages from −120 mV to +60 mV and held for 500 ms. Current amplitudes used for data analysis were taken from the last 100 ms of the voltage command jumps to ensure measurement of steady state current. Extracellular solutions used were either the NaNO_3_ NMDG-NO_3_, or ChNO_3_ solutions described above. Electrodes were pulled to a resistance of 0.5–2 MΩ and were filled with 3 M KCl. A 3 M KCl salt bridge was used for all experiments. All currents were recorded using pClamp10 software with on-line filtering at 1 kHz using a Bessel filter and digitized with a Digidata 1440A A/D converter (Molecular Devices) at 50 Hz.

### Whole-Cell Patch Clamp Recordings

Pipettes were pulled from borosilicate glass (Warner Instruments) and fire polished to a tip resistance of 1–5 MΩ. Pipette solutions contained 115 mM choline chloride, 2 mM MgCl_2_, 5 mM EGTA, and 10 mM HEPES. pH was adjusted to 7.3 with 8.6 M choline hydroxide (choline Cl solution). Extracellular solutions contained 140 mM NaSCN, 4 mM KCl, 2 mM MgCl_2_, 2 mM CaCl_2_, and 10 mM HEPES (NaSCN solution), or 144 mM KSCN, 2 mM MgCl_2_, 2 mM CaCl_2_, and 10 mM HEPES (KSCN solution). pH was adjusted to 7.4 with 10 N NaOH or KOH, respectively. Whole-cell recordings were made from tsA201 cells with an Axopatch 200B amplifier (Molecular Devices) in voltage-clamp mode 2–3 days after transfection. Series resistance compensation was set to at least 85% in all experiments. Signals were low-pass filtered at 5 kHz (8-pole Bessel; Warner Instruments) and collected at a sampling frequency of 10 kHz. Recordings from cells where series resistance exceeded 20 MΩ or holding current exceeded −100 pA at −70 mV in the presence of 100 μM DL-TBOA were excluded from analysis.

### Data Analysis and Statistics

Background currents were obtained from sham-injected oocytes for each extracellular condition and these currents were subtracted from the currents obtained in identical conditions in oocytes expressing the various EAAT isoforms. Data were analyzed with Prism v5 (Graphpad) or Sigmaplot (Jandel Scientific, San Rafael, CA, USA) and Clampfit 10 (Molecular Devices). Two-way ANOVAs comparing isoform vs. condition, unless otherwise noted, with *α* = 0.05 were used for all experiments. Bonferroni *post hoc* analysis tests were used to compare between groups. Asterisks indicate a significant difference between groups with * denoting a *p*-value of <0.05, ** indicating a *p*-value of <0.01 and *** indicating a *p*-value of <0.001.

## Results

### Neuronal and Glial EAATs Diverge by Their Na^+^-Dependence of Anion Channel Activation

**Figure 1:**
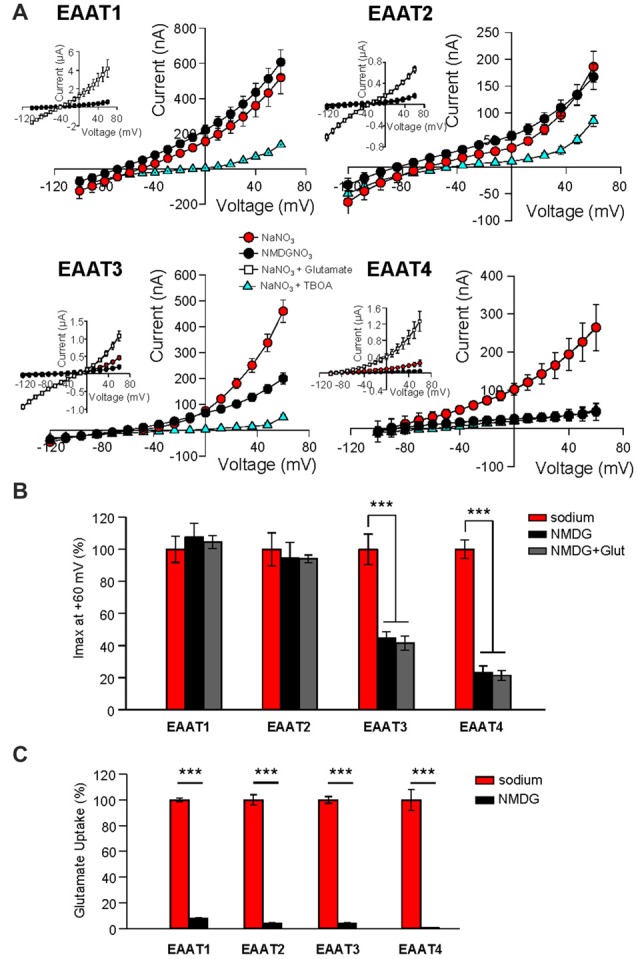
**Replacement of external Na^+^ with NMDG^+^ abolishes substrate independent anion permeation in excitatory amino acid transporters (EAATs) 3 and 4 but not EAATs 1 and 2. (A)** Averaged current-voltage relationships recorded from *Xenopus* oocytes expressing EAAT1–EAAT4. The conditions displayed are NaNO_3_–based solution (red circles), NMDG-NO_3_–based solution (black circles), NaNO_3_ + 100 μM DL-TBOA (blue triangles) and NaNO_3_ + 500 μM glutamate (open squares), and the insets illustrate the averaged current-voltage relationships obtained in the presence of saturating concentrations of L-glutamate (500 μM). **(B)** Bar graph representation of the current amplitudes at +60 mV in the same oocytes from **(A)**, the current amplitudes in NMDG-based solutions in the absence (black bars) and the presence (gray bars) of 500 μM glutamate are represented as percentage of the current in Na-based solutions (red bars). **(C)** Accumulation of 3,4-^3^H-L-glutamate in oocytes expressing EAAT1–EAAT4 in NaNO_3_-based solution (red) or NMDG-NO_3_-based solution (black) with 200 nM 3,4-^3^H-L-glutamate +10 μM glutamate added. Data in counts per minute (CPM) were normalized to transport activity in the NaNO_3_-based solution. The error bars represent the SEM for 12–25 oocytes per group from ≥3 frogs. Data in **(B,C)** were analyzed with a 2-way ANOVA comparing carrier vs. condition using a Bonferroni *post hoc* analysis.

The anion channel of EAAT4 is activated by binding of Na^+^, and open probability is further increased upon subsequent binding of glutamate, a process that also is Na^+^-dependent (Fairman et al., 1995; Melzer et al., 2003; Kovermann et al., 2010). Although it has been assumed that Na^+^ binding is required for the glutamate-independent anion channel activation of all EAAT isoforms, this assumption has not been tested. We therefore expressed EAATs 1–4 in *Xenopus* oocytes and measured the anion currents in extracellular NMDG-NO_3_ solution (Figure 1A, black circles, Figure 1B, black bars) and compared them with the current amplitudes obtained in extracellular NaNO_3_ solution (Figure 1A, red circles, Figure 1B, red bars, *n* = 12). The amplitudes of EAAT1-mediated NO_3_^−^ currents in Na^+^- and NMDG^+^–based solutions did not differ. Similarly, the replacement of extracellular Na^+^ with NMDG^+^ did not significantly alter NO_3_^−^ current amplitudes in EAAT2 expressing oocytes (Figures 1A,B, *n* = 15). In contrast, Na^+^ replacement with NMDG^+^ caused a significant reduction in currents measured in oocytes expressing EAAT3 (55.4% ± 3.9% reduction, *n* = 14) and EAAT4 (76.8% ± 4.0%, reduction, *n* = 18; Figures 1A,B). These currents can be attributed to the function of EAAT isoforms because application of a saturating concentration (500 μM) of L-glutamate increased current amplitudes in all cases (Figure 1A, insets, white squares, *n* > 13), and a saturating concentration (100 μM) of the specific, competitive inhibitor DL-TBOA reduced currents in oocytes expressing each isoform (Figure 1A, blue triangles, *n* > 12). Because the binding of glutamate to each of the EAAT carriers is dependent on the binding of 1 or 2 Na^+^ ions, replacing extracellular Na^+^ with NMDG^+^ should abolish substrate interactions. Consistent with the Na^+^-dependence of glutamate binding, co-application of glutamate with the NMDG-NO_3_ solution failed to significantly affect the current amplitude when compared to the application of NMDG-NO_3_ solution alone (Figure 1B, gray bars).

We also examined the effect of Na^+^ substitution with NMDG^+^ on radiolabeled substrate transport. 3,4-^3^H-L-glutamate uptake was significantly reduced in all isoforms (EAAT1: 92.2% ± 0.9%; EAAT2: 94.6% ± 2.0%; EAAT3: 94.6% ± 0.4%; and EAAT4: 103% ± 14.9% reduction; Figure 1C; *p* < 0.001 for all conditions). Thus, the glial isoforms EAAT1 and EAAT2, display a Na^+^-independent anion channel activity whereas anion channel activation of the neuronal isoforms, EAAT3 and EAAT4, exhibits strong Na^+^-dependence. However, all isoforms require Na^+^ to support glutamate translocation (Figure 1C).

**Figure 2:**
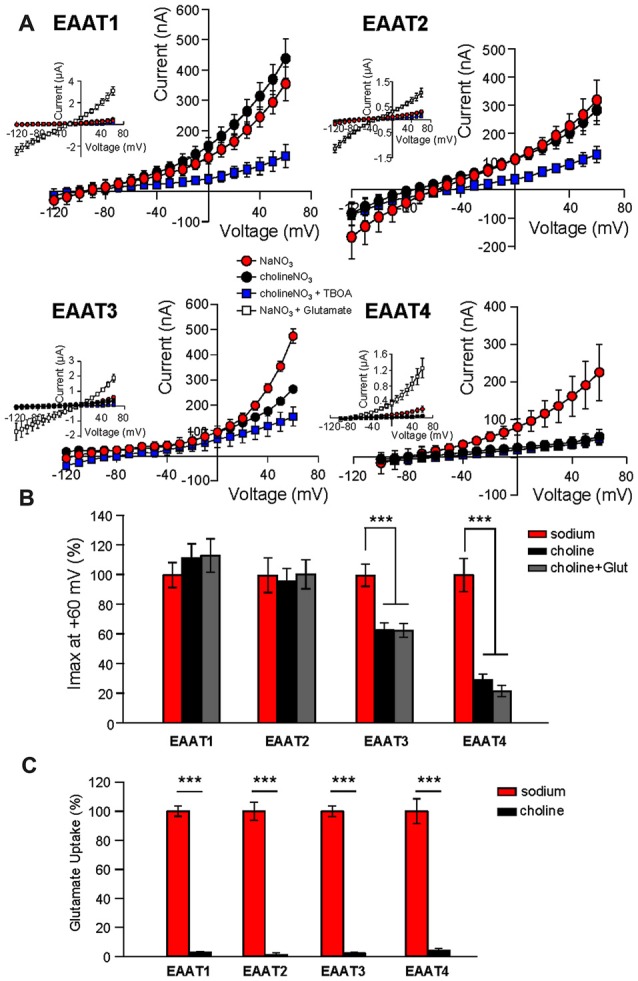
**Extracellular Na^+^ substitution with choline mimics results of substitution with NMDG^+^. (A)** Averaged current-voltage relationships from oocytes expressing EAAT1–EAAT4. The conditions displayed are NaNO_3_-based solution (red circles), cholineNO_3_-based solution (black circles), choline-based solution +300 μM DL-TBOA (blue squares), and NaNO_3_-based solution +500 μM glutamate (insets, open squares). **(B)** Bar graph representation of the current amplitudes at +60 mV in the same oocytes from **(A)**, the current amplitudes in choline-based solutions in the absence (black bars) and the presence (gray bars) of 500 μM glutamate are represented as percentage of the current in Na-based solutions (red bars). **(C)** Accumulation of 3,4-^3^H-L-glutamate in oocytes expressing EAAT1–EAAT4 in NaNO_3_-based solution (red) or cholineNO_3_-based solution (black) with 200 nM 3,4–^3^H-L-glutamate + 10 μM glutamate added. Data in CPM were normalized to transport activity in the NaNO_3_-based solution. The error bars represent the SEM for 12–23 oocytes per group from ≥3 frogs. Data in **(B,C)** were analyzed with a 2-way ANOVA comparing carrier vs. condition using a Bonferroni *post hoc* analysis.

Addition of glutamate to the NMDG-NO_3_ solution did not significantly affect current amplitudes at +60 mV (Figure 1B, gray bars), suggesting that NMDG^+^ does not bind to the transporter or at least not to the same site as Na^+^. However, it is possible that NMDG^+^ supports channel activity by interacting with EAAT1 and EAAT2 in an unforeseen manner. To test this possibility, we repeated the Na^+^ replacement experiments using choline as a substitute for Na^+^. As observed with the NMDG^+^ substitution experiments, replacement of extracellular Na^+^ with choline caused a significant reduction in channel activity in oocytes expressing EAAT3 (37.1% ± 4.8%, *n* = 12) or EAAT4 (70.8% ± 4.8%, *n* = 23) but not EAAT1 or EAAT2 (Figure 2A, black circles and Figure 2B, black bars, *n* > 14). In addition to choline not supporting the substrate-gated conductance, choline substitution also eliminated glutamate transport activity. Radiolabeled transport was significantly reduced in EAATs 1–4 (EAAT1: 96.4% ± 0.7%; EAAT2: 98.3% ± 3.3%; EAAT3: 95.7% ± 1.2%; and EAAT4: 90.9% ± 2.8% reduction; Figure 2C; *p* < 0.001 for all conditions, *n* > 12). To test whether the Na-independent currents observed in EAAT1 and EAAT2 expressing oocytes were actually mediated by the EAATs, we applied high concentrations of TBOA (300 μM) in the choline-based solutions. These currents observed in the absence of sodium in EAAT1 and EAAT2 were significantly blocked by TBOA, indicating they are in fact mediated by the transporters (Figure 2A, blue squares, *n* > 6).

### Na^+^ Mediated Channel Gating in EAAT3 and EAAT4 Is Concentration-Dependent

**Figure 3:**
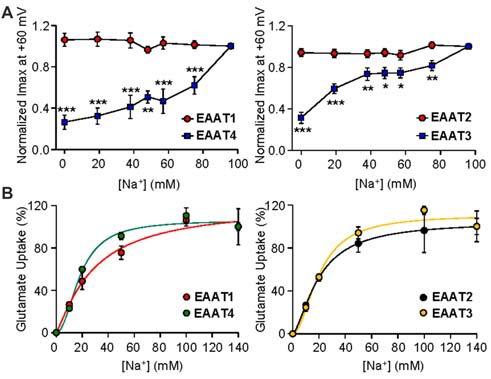
**Gating of the anion channel is Na^+^ concentration dependent only in neuronal EAATs. (A)** Averaged macroscopic currents amplitudes measured at +60 mV in response to perfusion of varying concentrations of Na^+^ in oocytes expressing either EAAT1 (red circles) or EAAT4 (blue squares; *left panel*) and in oocytes expressing EAAT2 (red circles) or EAAT3 (blue squares; *right panel*). Currents were normalized to the response to application of NaNO_3_ solution at +60 mV and represent the mean and SEM from 5 to 12 oocytes for each condition and ≥3 frogs. Data were analyzed by a 2-way ANOVA comparing carriers vs. Na^+^ concentration with a Bonferroni *post hoc* analysis. **(B)** Accumulation of 3,4-^3^H-L-glutamate by oocytes expressing EAAT1 (red circles) or EAAT4 (green circles; *left panel*), and from oocytes expressing EAAT2 (black circles) or EAAT3 (orange circles; *right panel*), in solution that contained 200 nM 3,4-^3^H-L-glutamate + 10 μM glutamate and the indicated concentration of sodium was replaced with equal molar concentrations of choline and transport was normalized to the maximum transport activity at 140 mM Na^+^. Data were fitted with a non-linear curve to obtain an EC_50_ and Vmax.

We next examined the nature of Na^+^ mediated channel gating in both glial and neuronal isoforms by assaying the relationship between Na^+^ concentration and channel activity. By systematically replacing increasing amounts of Na^+^ with equal molar concentrations of choline, we observed a significant dependence of EAAT4-mediated current on extracellular Na^+^ concentration (Figure 3A, left panel, blue squares). As Na^+^ concentration was decreased, EAAT4 displayed decrease in whole cell currents with a 73.5% ± 8.9% reduction of current amplitudes in 0 mM Na^+^ compared to 98 mM Na^+^ (*p* < 0.001). Currents in EAAT1-expressing oocytes were not significantly altered at any concentration of Na^+^ (Figure 3A, left panel, red circles). Comparing EAAT2 and EAAT3 demonstrates the same patterns of responses. Current amplitudes in EAAT3-expressing oocytes, in the absence of glutamate, are significantly reduced with systematic Na^+^ removal, yet EAAT2 current amplitudes do not significantly change at any concentration of Na^+^ (Figure 3A, right panel, red circles). EAAT3 currents were reduced by 68.6% ± 5.3% in 0 mM Na^+^ (Figure 3A right panel, blue squares, *p* < 0.001). Typically an estimate of Na^+^ affinity would be possible with the data generated by this type of assay, however, without a saturation of currents with increasing Na^+^ concentrations, a curve fit would not be accurate and therefore we did not attempt to estimate an affinity for Na^+^. It has been previously demonstrated that Na^+^ interactions are necessary to enable glutamate binding at the substrate site. In contrast to the lack of Na-dependence of the current amplitude for the glial isoforms, transport activity remained Na-dependent in all EAAT isoforms (Figure 3B). The sodium affinities obtained in our uptake assays were consistent with previous estimates of binding affinity for Na^+^ to outward facing transporters, which were 98 mM for EAAT2 (Wadiche et al., 1995b), 80 mM for EAAC1 (EAAT3; Watzke et al., 2001), and 42 mM for EAAT4 (Mim et al., 2005).

### Simultaneous Substitution of Intracellular and Extracellular Na^+^ Confirm the Na^+^-Independent Gating Mechanism for the Glial Transporters

**Figure 4:**
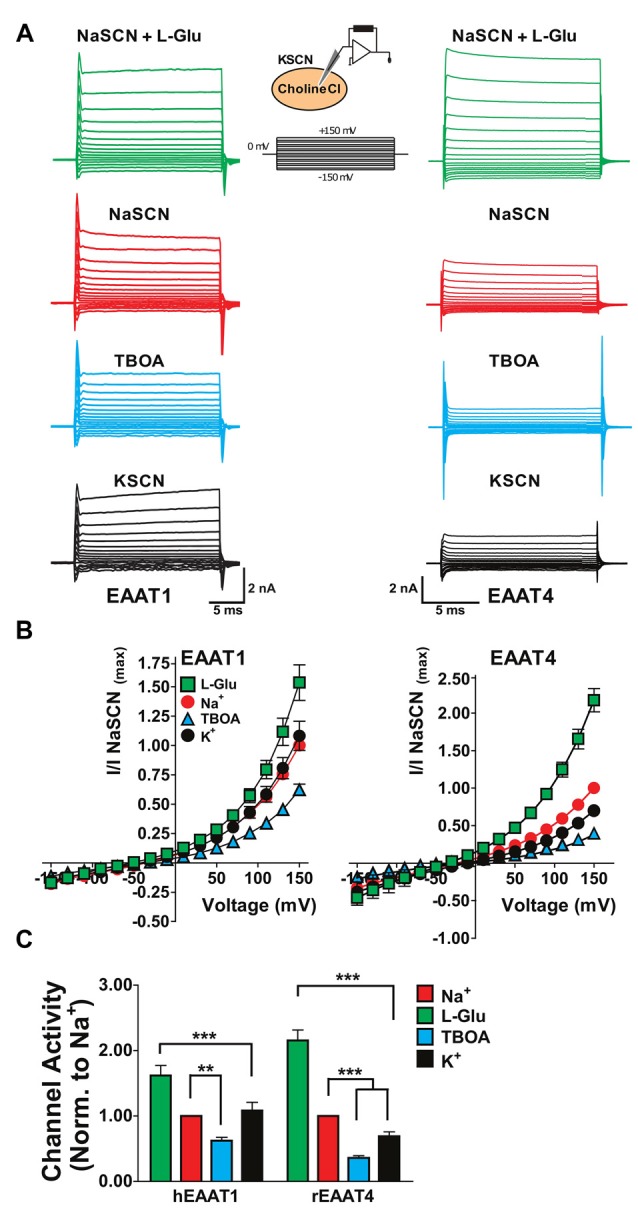
**Replacement of internal Na^+^ corroborates the Na^+^-independent anion channel activation of glial EAATs. (A)** Representative current responses to voltage commands in tsA201 cells expressing EAAT1 (left) or EAAT4 (right) using whole-cell patch clamp recordings.** (B)** Whole-cell patch clamp recordings of tsA201 cells transiently expressing EAAT1 (*left*) and EAAT4 (*right*). Cells were dialyzed with a cholineCl pipette solution as described in the experimental procedures. Extracellular solutions used were NaSCN solution + 500 μM L-glutamate (green squares), NaSCN solution (red circles), NaSCN solution + 100 μM DL-TBOA (blue triangles), and KSCN solution (black circles). Currents were normalized to the response elicited by application of NaSCN solution alone at +150 mV and represent the mean and SEM of 3–7 cells. **(C)** Quantitation of current amplitudes at +150 mV from experiments described in **(A,B)**. Extracellular conditions displayed are NaSCN (Na^+^ red), NaSCN + 500 μM glutamate (green), NaSCN + 100 μM DL-TBOA (blue), and KSCN solution (K^+^, black). Data were analyzed by a 2-way ANOVA comparing carrier vs. condition with a Bonferroni *post hoc* analysis.

One explanation for the substrate-independent channel activity in the absence of extracellular Na^+^ in EAATs 1 and 2 (Figures 1–3) could be that, with extracellular replacement of Na^+^, intracellular Na^+^ can bind to the transporter and gate the channel. To explore this possibility, we used whole-cell patch clamp recordings to compare the currents associated with the glial EAAT1 and the neuronal EAAT4 in cells dialyzed with a Na^+^-free solution. Initially, NaSCN solution was perfused extracellularly with or without saturating concentrations of either L-Glu or TBOA to demonstrate normal function of the EAAT transporters in this system. To compare currents amplitudes in the presence or absence of intracellular binding of Na^+^ to the EAATs, NaSCN solutions were replaced extracellularly with KSCN which would increase the relative proportion of inward-facing transporters and current amplitudes were compared with cholineCl solution substituted intracellularly. Under these conditions EAAT1 current amplitudes were not significantly altered (Figures 4A (black), 4B (black circles), 4C (black)) compared to current amplitudes with an intracellular Na^+^-free solution and extracellular NaSCN solution (Figures 4A (red), 4B (red circles), 4C (red)). However, the amplitude of EAAT4 currents were reduced by 27.6% ± 0.8% (*p* < 0.001, Figures 4A–C). These results rule out the possibility that intracellular Na^+^ is sufficient to activate the EAAT1 channel and further support the contention that the substrate-independent channel activity in the glial transporters is Na^+^-independent.

Taken together our results indicate that although substrate transport is consistently Na^+^-dependent in all EAATs, the substrate-independent anion channel activity shows a differential requirement for Na^+^ between glial and neuronal isoforms. In the neuronal isoforms, the substrate-independent currents are sodium-dependent as previously observed, whereas the glial isoforms display similar current amplitudes in the absence or the presence of sodium, consistent with the existence of Na^+^-independent conducting state(s) in EAATs 1 and 2.

## Discussion

Here we demonstrate a difference in the sodium requirements for anion channel gating between neuronal and glial EAAT isoforms. The ability of EAATs to mediate an anion flux in the absence of glutamate, classically termed the “leak” conductance, is well established. Until now, this substrate-independent conductance was thought to be Na^+^-dependent in all isoforms. Our data indicate that Na^+^-binding to EAAT3 and EAAT4 is necessary for channel opening, but EAATs 1 and 2 mediate a substrate- and Na^+^-independent anion flux which is comparable in magnitude to that observed in the presence of sodium. Initial studies into the EAAT-mediated anion conductance displayed the loss of a tonic current in Müller glial cells when extracellular Na^+^ was replaced by choline (Schwartz and Tachibana, 1990). Alternatively, the presence of the tonic or substrate-independent currents in Na^+^ alone (glutamate free) conditions was proposed by Vandenberg et al. (1995) and further validated using non-transported inhibitors (Arriza et al., 1997; Bergles and Jahr, 1997; Wadiche and Kavanaugh, 1998; Grewer et al., 2000). Studies which described the Na^+^-dependence of these anion currents were, however, limited to neuronal EAAT3 and EAAT4 isoforms (Watzke et al., 2001; Mim et al., 2005; Torres-Salazar and Fahlke, 2006) or Müller glial cells from salamander (Schwartz and Tachibana, 1990), the latter of which displays some disparate properties from the mammalian EAATs (Eliasof et al., 1998). A recent study conducted by Fahlke’s group examining the regulation of EAAT2 channel activation by C-terminal domains, suggested the existence of a Na^+^-independent channel activity, leading the authors to speculate that substrate and Na^+^-independent anion conducting states might be a unique feature of this isoform (Leinenweber et al., 2011). Here, we demonstrate that substrate and Na^+^-independent conducting states are an intrinsic property of both glial EAAT isoforms not observed in their neuronal counterparts.

Glial EAATs mediate the majority of glutamate transport in the brain. EAAT1 (GLAST), is the major glutamate transporter in the cerebellum (Lehre and Danbolt, 1998) and the retina (Rauen et al., 1999). EAAT2 (GLT-1 in rodents) shows high levels of expression in the glial membranes throughout the brain (Lehre and Danbolt, 1998; Holmseth et al., 2012). Strong evidence supports the hypotheses that neuronal transporters such as EAAT3 and EAAT4 limit activation of peri- or extra-synaptic receptors through rapid buffering of synaptically released glutamate (Tong and Jahr, 1994; Diamond and Jahr, 1997; Diamond, 2001; Scimemi et al., 2009). EAATs have a turnover rate on the order of 1–100 cycles per second across the isoforms (Wadiche et al., 1995b; Grewer et al., 2000). One of the early kinetic models of the glutamate transport cycle described a 15 state transport cycle with eight anion conducting states in GLT-1 (rat EAAT2). These conducting states were limited to Na^+^-bound and Na^+^- and glutamate-bound states, in the outward facing conformations. Recently, experimental data were combined with simulations to further refine our knowledge of the relationship between conducting states and transporter conformations in EAAT4 (Machtens et al., 2011b). These data support the hypothesis that there are additional conducting states, which are associated with conformations not previously thought to mediate channel activity. Indeed an intermediate-outward facing crystal structure of the excitatory amino acid transporter from *Pyrococcus horikoshii* (Glt_*Ph*_; Verdon and Boudker, 2012) displayed a cavity that the authors proposed as a putative anion conduction pathway. This structure provides a snapshot of a stabilized conformation and does not capture all the various stable and intermediate conformational states that the carrier may adopt through the transport cycle. Machtens et al. (2015) used a combination of molecular dynamic simulations, electrophysiological recordings and fluorescence spectroscopy, to support mediation of anion conductance by intermediate states during the translocation cycle, similar to a state observed by Verdon and Boudker (2012). It is now clear that there are several intermediate states that comprise both small and large movements which can lead to anion permeation. Indeed, Na^+^ interactions alone have been shown to mediate small conformational changes (Watzke et al., 2001; Koch and Larsson, 2005). Highlighting the complex nature of channel gating in EAATs, we recently identified a point mutation in EAAT1 (R388D/E) and EAAT4 (R410E) that results in a constitutively open anion channel and favors a conformation resembling an intermediate state (Torres-Salazar et al., 2015). The difference in the motions between the glial and neuronal carriers remains to be resolved. However, we can now hypothesize that these differences in the Na^+^-dependence of low open probability conducting states are likely limited to early intermediates in either the inward or outward halves of the transport cycle.

It is unclear what role the neuronal or glial EAAT-mediated channel activity plays in glutamatergic neurotransmission. The role of the anion channel in regulating cellular activity has been supported by EAAT5-mediated regulation of rod bipolar cells in the retina (Veruki et al., 2006). Recent studies suggest that the anion channels associated with the glial glutamate transporters, EAAT1 and EAAT2, are involved in regulation of glial intercellular chloride concentrations, potentially influencing crosstalk between glutamatergic and GABAergic neurotransmission (Untiet et al., 2017). At this point, all experiments testing the ability of EAAT-mediated anion currents to affect cellular activity have always been performed in the presence of glutamate and no data exist that support a role for the EAAT-mediated regulation of cellular activity in the absence of glutamate. Thus it is challenging to speculate on the impact of these low open probability conducting states in the presence of Na^+^ alone (EAAT3 and EAAT4) or in the unbound transporter (EAATs 1 and 2). The additional knowledge provided in this study will assist in generating more meaningful hypotheses for future studies.

## Author Contributions

CDB, SGA designed the experiments. CDB, DT-S, JEB, NGG, ADG-S acquired the data. CDB, DT-S, JEB, NGG, JWJ, ADG-S, and SGA analyzed the data. CDB, DT-S and SGA wrote the manuscript. JEB, NGG, and JWJ critically revised the manuscript.

## Conflict of Interest Statement

The authors declare that the research was conducted in the absence of any commercial or financial relationships that could be construed as a potential conflict of interest.
